# Folic Acid Supplementation Mitigates Alzheimer's Disease by Reducing Inflammation: A Randomized Controlled Trial

**DOI:** 10.1155/2016/5912146

**Published:** 2016-06-02

**Authors:** Hui Chen, Shuai Liu, Lu Ji, Tianfeng Wu, Yong Ji, Yuying Zhou, Miaoyan Zheng, Meilin Zhang, Weili Xu, Guowei Huang

**Affiliations:** ^1^School of Nursing, Tianjin Medical University, Tianjin, China; ^2^Department of Neurology, Huanhu Hospital, Tianjin, China; ^3^Department of Nutrition and Food Science, School of Public Health, Tianjin Medical University, Tianjin, China; ^4^Metabolic Diseases Hospital & Tianjin Institute of Endocrinology, Tianjin, China; ^5^Aging Research Centre, Department of Neurobiology, Care Sciences and Society (NVS), Karolinska Institutet, Stockholm, Sweden

## Abstract

*Background/Aims*. Low serum folate levels can alter inflammatory reactions. Both phenomena have been linked to Alzheimer's disease (AD), but the effect of folic acid on AD itself is unclear. We quantified folate supplementation's effect on inflammation and cognitive function in patients with AD over the course of 6 months.* Methods*. Patients newly diagnosed with AD (age > 60 years; *n* = 121; mild to severe; international criteria) and being treated with donepezil were randomly assigned into two groups with (intervention group) or without (control group) supplemental treatment with folic acid (1.25 mg/d) for 6 months. The Mini-Mental State Examination (MMSE) was administered to all patients at baseline and follow-up, and blood samples were taken before and after treatment. We quantified serum folate, amyloid beta (A*β*), interleukin-6 (IL-6), tumor necrosis factor *α* (TNF*α*), plasma homocysteine (Hcy), S-adenosylmethionine (SAM), S-adenosylhomocysteine (SAH), and the mRNA levels of presenilin (PS), IL-6, and TNF*α* in leukocytes. Data were analyzed using a repeated-measures mixed model.* Results*. The mean MMSE was slightly increased in the intervention group compared to that in the control group (*P* < 0.05). Posttreatment, plasma SAM and SAM/SAH levels were significantly higher (*P* < 0.05), while A*β*_40_, PS1-mRNA, and TNF*α*-mRNA levels were lower in the intervention group than in the control group (*P* < 0.05). The A*β*_42_/A*β*_40_ ratio was also higher in the intervention group (*P* < 0.05).* Conclusions*. Folic acid is beneficial in patients with AD. Inflammation may play an important role in the interaction between folic acid and AD. This trial is registered with clinical trial registration number ChiCTR-TRC-13003246.

## 1. Introduction

As the world's population ages, it is becoming increasingly difficult to control the growing burden of dementia. This is especially true for Alzheimer's disease (AD), the most common type of dementia in the elderly [1]. In China, it is estimated that the incidence of AD had reached 6.25 cases per 1000 person-years in 2010 [2]. AD is an age-related neurodegenerative disease, characterized by memory loss and cognitive decline, which leads to death within 7–10 years of diagnosis [3]. The mechanisms underlying the degenerative processes remain unclear, although epigenetic and neuroinflammatory disturbances, in addition to the apparent contribution of aging itself, have been implicated in AD [4, 5].

As mentioned, inflammatory processes are being investigated in the pathological mechanisms of AD and mild cognitive impairment (MCI) [5]. Low folate levels impair vitamin B_12_ absorption, which in turn may lead to an inflammatory state, and thus explain the relationship between both of these compounds and AD risk [6]. Indeed, studies have reported that folate levels are lower in patients with AD than in normal controls [7–9].

Effective treatments for AD are also relatively few and only mitigate the progression of the disease. To improve cognitive function, many clinicians recommend that patients with AD take folate and other B vitamin supplements, but the effectiveness of these remains controversial [10, 11]. We have previously reported that the risk of AD is increased in individuals with low serum folate levels [7, 12]. Here, we further explore the role of inflammation in mediating the beneficial effects of folic acid on newly diagnosed AD patients with no previous folate supplementation.

## 2. Materials and Methods

### 2.1. Participants

The Department of Nutrition and Food Science, School of Public Health, Tianjin Medical University, and Department of Neurology, Huanhu Hospital, Tianjin, designed the TFA-AD trial. Participants were recruited between November 2013 and October 2014 by neurologists at the Neurology Central Hospital of Tianjin.

We recruited participants with a new diagnosis of possible or probable AD of mild to moderate severity, defined as a Mini-Mental State Examination (MMSE) total score between 3 and 26 [13], and those who were currently taking donepezil. Individuals who met the following criteria were included in the study: age 40–90 years; Hachinski Ischemic Scale score <4; Hamilton Depression Rating Scale score <7.0; MMSE score 3.0–26.0 points; activities of daily living (ADL) [14] score ≥22.0; cerebral computed tomography or magnetic resonance imaging showing varying degrees of ventricular dilatation; medial temporal lobe and hippocampal atrophy, widening of the cerebral sulci, and other cortical atrophies; and the absence of encephalopathy, which can appear clinically similar to AD. We excluded patients who had taken the prescribed daily intake of vitamins B, C, and E within the month before baseline and those who had regularly taken B vitamin supplements within the 6 months before baseline. All participants had normocytic anemia (mean corpuscular volume: 82–95 FL) and normal renal function (creatinine clearance: 80–120 mL/min). All participants or their surrogates provided written informed consent. Written consent for collection of caregiver data was also obtained from the participants' designated caregivers.

### 2.2. Interventions

This was a single-center, single blind, randomized controlled trial (RCT) to assess whether folic acid could effectively delay clinical progression in patients with AD. Patients were randomly assigned in a 1 : 1 ratio to either the treatment or the control group, using randomization software. At the baseline visit, each investigator sequentially assigned a randomization number to each patient. No individual patient randomization code was revealed during the trial. Monitors from the Clinical Research Organization and the sponsor visited investigators regularly to conduct quality control checks and ensure the validity and accuracy of recording and overall adherence to the study protocol. Data were entered by double entry, and computerized checks were performed to ensure the consistency of data.

All participants took donepezil as basic routine therapy. The initial dose of donepezil was 5 mg daily and was increased to 10 mg after one month. Participants having difficulty tolerating the higher dose of donepezil were excluded by the investigator. Although the optimal folic acid dose needed to improve cognitive function is still not known, Durga et al. tested the effects of 800 *μ*g/d folic acid supplementation on cognitive function in older adults over a period of 3 years [11]. McMahon et al. reported the effects of 2 years of 1 mg/d folic acid supplementation on healthy older people [10]. Considering previous durations and dosages, this study examined 6 months of 1.25 mg/d folic acid supplementation in patients with AD. Participants randomized into the folic acid supplementation group received 1.25 mg folic acid daily during or immediately after a meal, in tablet form (Beijing Scrianen Pharmaceutical Co. Ltd., China; 5 mg/tablet; state medical permit number H10970079), for the entire six-month period. These folic acid supplements are available by prescription in China. Adherence was encouraged and monitored throughout the trial by telephone. Clinical outcomes were assessed at baseline and at 6-week intervals.

Each patient had a responsible caregiver, and both participants and their caregivers provided written informed consent. The study was conducted in accordance with the Declaration of Helsinki and the ICH-GCP as appropriate to nutritional products and approved by the Ethics Committee of the Tianjin Health Service.

### 2.3. Outcome Measures

Primary outcome measures included the differences between the baseline and 6-month scores on the MMSE and ADL and the levels of amyloid beta A*β*_40_, A*β*_42_, A*β*_42_/A*β*_40_, amyloid precursor protein mRNA (APP-mRNA), PS1-mRNA, PS2-mRNA, IL-6-mRNA, and TNF*α*-mRNA. Secondary outcome measures included the differences between the baseline and 6-month values of SAM, SAH, SAM/SAH, and Hcy.

### 2.4. Blood Sample Collection

Fasting venous blood was collected into two tubes for processing. A tube containing coagulant was centrifuged at 3,000 g for 10 min, and the serum was drawn off and stored frozenly at −80°C. Serum samples were divided for the subsequent measurement of (1) folate and vitamin B_12_ levels and (2) A*β*_40_, A*β*_42_, IL-6, and TNF*α* levels. Another tube containing ethylenediaminetetraacetic acid (EDTA) was immediately centrifuged at 2,500 g for 10 min at 4°C. Plasma samples were decanted and stored frozenly at −80°C for the subsequent measurement of Hcy, S-adenosylmethionine (SAM), and S-adenosylhomocysteine (SAH). The white blood cells were obtained and stored frozenly at −80°C for the subsequent measurement of PS1-mRNA, PS2-mRNA, IL-6-mRNA, and TNF*α*-mRNA.

### 2.5. Measurement of Biochemical Parameters

Serum folate and vitamin B_12_ levels were measured using an automated immunoassay analyzer (Abbott AxSYM system, Abbott Laboratories, Abbott Park, IL, USA) [15, 16]. Plasma levels of Hcy, SAM, and SAH were analyzed via high-performance liquid chromatography (HPLC), as described by Poirier et al. [17], and quantified relative to standards obtained from Sigma Chemical Co. (St. Louis, MO, USA). AD gene-related mRNAs were analyzed using real-time polymerase chain reaction (RT-PCR). The interassay coefficients of variation ranged from 3 to 10%, within an appropriate range unlikely to induce statistical errors.

A*β*_40_, A*β*_42_, IL-6, and TNF*α* in the patients' blood were quantified with a standard ELISA kit (Biosources, Camarillo, CA). Briefly, frozen samples were sequentially extracted via a two-step extraction process (sonication in 2% SDS and 70% formic acid). After sonication, the samples were centrifuged at 100,000 g for 1 h at 4°C, and the supernatant was decanted. The serum was then sonicated with the formic acid solution. A*β*_40_, A*β*_42_, IL-6, and TNF*α* were quantified separately, using an ELISA kit specific for each amyloid fragment in accordance with the manufacturer's instructions.

Real-time PCR was performed to analyze the different AD gene-related mRNA levels. The total RNA in the blood was reverse-transcribed to cDNA using RNase Inhibitor, dNTP, RT buffer, oligo(dT), DEPC H_2_O, and AMV (Takara). The mixture was incubated at 42°C for 50 min, 99°C for 5 min, and 5°C for 5 min to generate a cDNA library. Real-time PCR was subsequently performed using a LightCycler 480 SYBR Green I Master system (Roche, USA) according to a standardized protocol. The reactions were incubated at 95°C for 5 min, followed by 45 cycles at an interval of 10 s at 95°C, 5–20 s at 72°C, and 5 s at 95°C and an interval of 60 s at 60°C. Data were analyzed by 2^−ΔCT^. The primer sequences of the internal control gene and the target genes are listed in Table 1.

### 2.6. Sample Size

**Figure 1:**
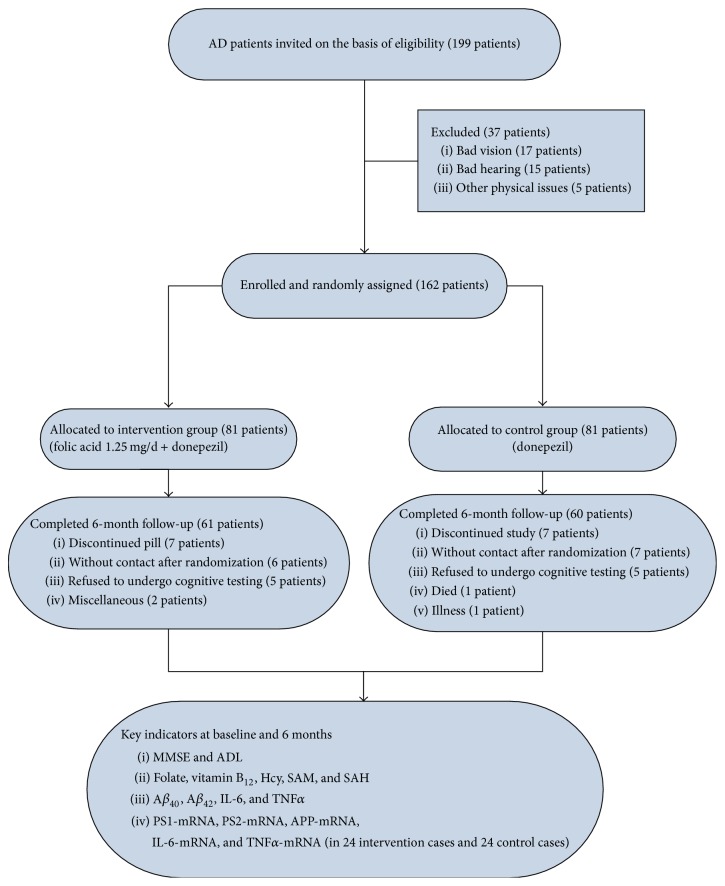
Study participant flow diagram from the initial contact through the completion of the study.

Assuming a hypothetical effect size of 0.6, a sample size in each intervention group provided 90% power at a 2-sided *α* level of 0.05, based on a 2-sample *t*-test for change between baseline and 6 months in both MMSE and ADL scores [18]. With allowance for a dropout rate of 25% [19], each intervention group needed at least 75 patients at baseline and 60 patients after 6 months. We started with 81 patients at baseline and concluded with 61/60 patients after 6 months in each intervention group (Figure 1).

### 2.7. Statistical Analysis

All statistical tests were performed using SPSS for Windows v.15.0 (SPSS Inc., Chicago, IL, USA). Data are presented as means ± standard deviations (SD), medians (quartiles), or proportions. Statistical significance was set at *P* < 0.05. Comparisons between categorical variables were conducted using chi-square tests. Quantitative variables were checked for normality using histograms and quartile-quartile plots. Age at diagnosis, education (years), body mass index (BMI), folate, vitamin B_12_, MMSE, and ADL were normally distributed and thus evaluated using two-tailed Student's *t*-tests at baseline. Hcy, SAM, SAH, and SAM/SAH were normally distributed after square root transformation. A*β*_40_, A*β*_42_, A*β*_42_/A*β*_40_, and AD gene-related mRNA levels were normally distributed after logarithmic transformation.

Repeated-measures ANOVAs were used to evaluate the effects of folic acid and control interventions on biomarker and cognitive performance over 6 months, in which time was treated as a categorical variable and represented by dummies. Data are mean unadjusted scores plus standard deviations, with *ηp* ^2^ and *P* values from repeated-measures ANOVAs that included the time-treatment interaction.

Patients who had one assessment after baseline were included in the intention-to-treat (ITT) efficacy analysis.

## 3. Results

### 3.1. Characteristics of the Study Participants

The flow of participants through the study is shown in Figure 1. Of the 199 initial patients with AD, 37 were excluded for bad vision, bad hearing, or other physical issues, leaving 162 patients with AD to be randomly allocated between the groups. Of these, 61 patients in the folate supplementation group were excluded as follows: 7 discontinued the supplement, 6 could not be contacted after randomization, 5 refused to undergo cognitive testing, and 2 were excluded for other reasons. In the control group, 60 patients were excluded as follows: 7 discontinued the study, 7 could not be contacted after randomization, 5 refused to undergo cognitive testing, 1 died, and 1 became ill. No relevant differences among the groups were detected at baseline.

A summary of participants' characteristics at baseline is shown in Table 2. Demographics, lifestyle, family history, comorbidities, and current AD-related medications were not significantly different between the two groups. Furthermore, the intervention (folate supplementation) and control groups had similar levels of folate and vitamin B_12_ (*P* > 0.05).

### 3.2. Methionine-Related Metabolites in Serum

**Figure 2:**
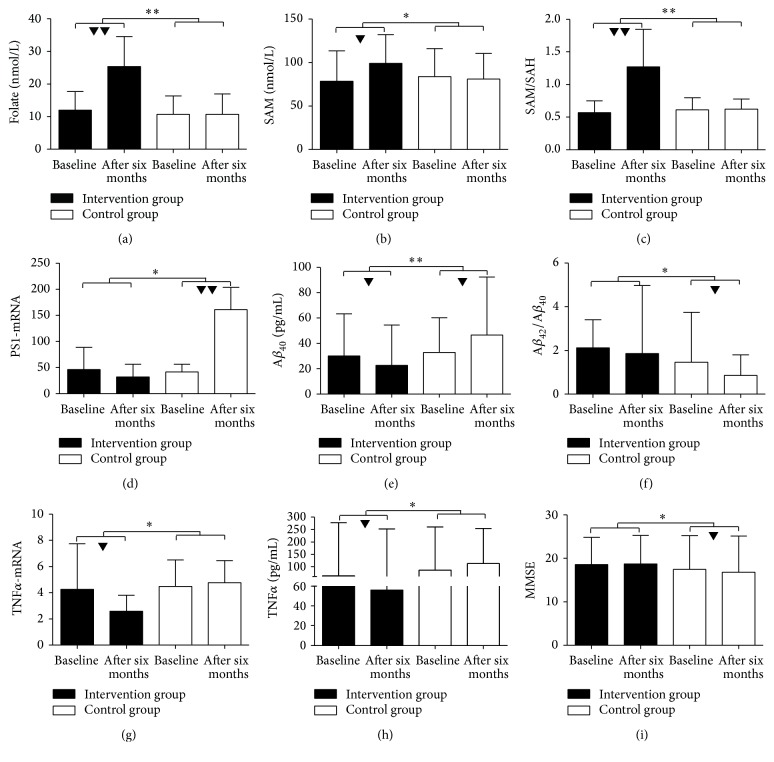
Changes in peripheral biochemical factors (folate, SAM, SAM/SAH, TNF*α*-mRNA, TNF*α*, PS1-mRNA, A*β*_40_, and A*β*_42_/A*β*_40_) and MMSE at baseline and 6 months, in the intervention and control groups. Results are given as mean ± SD or median with interquartile range. Figures indicate a significant difference from the baseline to six months, between intervention group and control group by analysis of repeated-measures ANOVA (^▼, *∗*^ *P* < 0.05; ^▼▼, *∗∗*^ *P* < 0.01).

ITT analyses showed that, over 6 months, participants in the intervention group had a significantly greater increase in serum folate than those in the control group (*P* = 0.001, *ηp* ^2^ = 0.937). Plasma SAM (*P* = 0.012, *ηp* ^2^ = 0.712) and SAM/SAH levels (*P* = 0.000, *ηp* ^2^ = 0.999) demonstrated greater increases in the intervention group than in the control group. However, there were no significant differences in levels of vitamin B_12_, Hcy, and SAH (Table 3, Figure 2).

### 3.3. A*β*, IL-6, and TNF*α* ELISAs

ELISAs of serum amyloid load and ITT analyses demonstrated that A*β*_40_ was significantly lower in the intervention group than in the control group (*P* = 0.003, *ηp* ^2^ = 0.843). The A*β*_42_/A*β*_40_ ratio was significantly higher in the intervention group (*P* = 0.029, *ηp* ^2^ = 0.594). TNF*α* was significantly lower in the intervention group versus the control group (*P* = 0.021, *ηp* ^2^ = 0.638) (Table 3, Figure 2).

### 3.4. RT-PCR of Alzheimer's Disease-Related mRNA

Of the Alzheimer's disease-related mRNA species in the blood, PS1-mRNA was significantly lower in the intervention group compared to the control group (*P* = 0.017, *ηp* ^2^ = 0.676). TNF*α*-mRNA was also significantly lower in the intervention group versus the control group (*P* = 0.044, *ηp* ^2^ = 0.527). However, there were no significant differences in PS2-mRNA or IL-6-mRNA (Table 4, Figure 2).

### 3.5. Cognitive Function

Repeated-measures analyses revealed that, over 6 months, the MMSE scores were significantly higher in the intervention group than in the control group (*P* = 0.041, *ηp* ^2^ = 0.538). However, there was no significant difference in ADL scores (Table 3, Figure 2).

## 4. Discussion 

In this study, the patients with AD were not exposed to folic acid derived from fortification of food items. In western countries, folic acid fortification programs may mask the relationship between folate status and cognitive decline, as suggested by the limited and inconclusive findings of previous studies. The level of folate intake in China is usually 30–40% lower than the recommended dietary allowance [20] because of the lack of a governmental folic acid fortification program and traditional cooking methods that may cause folate to be lost from cooked vegetables. We treated patients with AD with or without folic acid (1.25 mg/d) for 6 months. All patients were to maintain stable donepezil therapy at the entry dose prescribed by the patient's physician for the duration of the study, and if donepezil therapy was discontinued, patients were excluded from the study.

### 4.1. Effect of Folic Acid Supplementation on Levels of Inflammatory Cytokines and Cognitive Impairment/Dementia

Many clinicians recommend that patients with AD take B vitamin supplements, in hopes of maintaining cognition. Peripheral inflammation is an important and potentially modifiable condition for patients who complain of changes in cognitive performance. Several population-based studies have shown that peripheral inflammatory cytokines, especially IL-6, TNF*α*, and IL-1*β*, are correlated with the risk of dementia [21, 22]. Increased peripheral cytokine levels have been associated with disrupted hippocampal stem cell function, reduced hippocampal volume, and reduced memory performance [23–25]. The present study also supports the role of inflammation in increasing the risk of developing dementia. Our data indicated that TNF*α* protein (interaction effect: *P* = 0.021, *ηp* ^2^ = 0.638) and TNF*α*-mRNA (interaction effect: *P* = 0.044, *ηp* ^2^ = 0.527) were significantly lower in the intervention group versus the control group.

The neuropathological features of AD include extracellular senile plaques composed primarily of A*β* [26–28]. The two major forms of A*β* are A*β*_40_ and A*β*_42_. In a normal individual, the majority of A*β* produced is A*β*_40_, whereas mutations causing familial AD lead to either increased A*β*_42_ production or reduced A*β*_40_ without a concomitant increase in A*β*_42_ production (i.e., an increased A*β*_42_/A*β*_40_ ratio) [29, 30]. This differs from our results, which showed a decreased A*β*_42_/A*β*_40_ ratio (interaction effect: *P* = 0.029, *ηp* ^2^ = 0.594), although, in our patients, those with a family history of AD accounted for just 26.23% of the intervention group and 25% of the control group. We found that A*β*_40_ (interaction effect: *P* = 0.003, *ηp* ^2^ = 0.843), but not A*β*_42_, was significantly reduced in the serum of the intervention group versus the control group. We also found that PS1-mRNA (interaction effect: *P* = 0.017, *ηp* ^2^ = 0.676) was significantly lower in the intervention group versus the control group. Thus, our study participants were mostly sporadic cases, with potentially different underlying mechanisms of pathology. The results of other studies support our findings. For example, in one study, the A*β*_42_/A*β*_40_ ratio in cerebrospinal fluid (CSF) samples from patients with AD was significantly (approximately 38%) lower than in age-matched controls, and a significant linear correlation was detected between A*β*_40_ concentration and MMSE scores [31]. In patients with MCI, CSF A*β*_42_ concentrations and A*β*_42_/A*β*_40_ ratio at baseline were significantly lower in those patients who subsequently developed AD than in those who were cognitively stable or who developed other forms of dementia [32]. Finally, a lower plasma A*β*_42_/A*β*_40_ ratio is also associated with greater cognitive decline among nondemented elderly individuals [33].

### 4.2. Effect of Folic Acid Supplementation on Levels of SAM and SAM/SAH

Folate is an essential vitamin that is involved in various biochemical reactions including the SAM metabolic cycle (i.e., one-carbon metabolism). There is an inverse relationship between folic acid and SAM concentrations throughout the one-carbon cycle. In this trial, daily use of folic acid supplements increased plasma SAM (interaction effect: *P* = 0.012, *ηp* ^2^ = 0.712) and SAM/SAH (interaction effect: *P* = 0.000, *ηp* ^2^ = 0.999) concentrations in elderly individuals with AD. SAM itself appears to be altered in some neurological disorders, including AD [34–36]. For example, Trolin and coworkers treated patients with AD with vitamin B_12_, SAM, and folate for 6 months, causing a significant decrease in Hcy levels [37], while Bottiglieri et al. [38] noted that oral SAM treatment (1200 mg daily) for 4 to 8 months was associated with a significant increase in CSF SAM, indicating that oral SAM does cross the blood-brain barrier.

### 4.3. Effect of Folic Acid Supplementation on Levels of Inflammatory Cytokines and Cognitive Impairment/Dementia Mediated by SAM

Inflammation is one of the earliest neuropathological events in AD. TNF*α* plays a critical role in the development of inflammation-induced AD. SAM has been associated with the proinflammatory response in vitro and in animal models and in humans. Declining or already low concentrations of folate may exert detrimental effects on cognitive function through systemic inflammation.

SAM has been shown to lower lipopolysaccharide- (LPS-) induced expression of the proinflammatory cytokine TNF*α* and increase the expression of the anti-inflammatory cytokine IL-10 in macrophages. Relative to controls, treatment with 500 *μ*mol/L SAM caused a significant decrease in TNF*α* expression and increase in IL-10 expression in human monocytic THP1 cells [39]. Inhibition of LPS-induced PDE4B2 upregulation and increased cAMP-dependent protein kinase A activation are significant mechanisms contributing to the anti-TNF effect of SAM [40]. SAM supplementation can also decrease oxidative stress by upregulating glutathione synthesis, reducing inflammation via downregulating TNF*α*, upregulating IL-10 synthesis, increasing the ratio of SAM to SAH, inhibiting the apoptosis of normal hepatocytes, and stimulating the apoptosis of liver cancer cells. Folate deficiency may accelerate or promote alcoholic liver disease (ALD) by increasing hepatic homocysteine and SAH concentrations, decreasing hepatic SAM and glutathione concentrations and the SAM-SAH ratio, and inhibiting apoptosis. In addition, folate deficiency has been found to increase Hcy and SAH concentrations [41].

### 4.4. The Combination of Folic Acid and Donepezil as an Improved Therapy for AD

After 6 months, significant improvement in the MMSE scores (i.e., cognitive function) (interaction effect: *P* = 0.041, *ηp* ^2^ = 0.538) was noted in the intervention group versus the control group (*P* = 0.041, *ηp* ^2^ = 0.538). There is evidence suggesting that donepezil/folate combination therapy could be beneficial. Sharma and Singh found that sodium diethyldithiocarbamate trihydrate, folacin, and donepezil significantly improved hyperhomocysteinemia/cholesterol-induced impairment of learning, memory, endothelial dysfunction, and changes in various biochemical parameters in patients with vascular dementia [42]. Connelly et al. also found significant improvement when an acetylcholinesterase inhibitor (AChEI) was combined with folate [43]. Though the mechanism is unclear, the benefits may be related to coaction on methylation-controlled A*β* production and modification of brain gene expression [44]. Folic acid deficiency may compromise antioxidative activity at multiple levels [44] and may elevate Hcy. Hcy stimulates NMDA receptors, leading to increased reactive oxygen species, which in turn stimulate hyperphosphorylation of tau [45]. It is particularly intriguing that folic acid could compensate for the probable toxicity of cholinesterase [46] as well. Therefore, we hypothesize that there is a possible synergistic effect of folic acid with donepezil, a combination therapy that is increasingly used in clinical practice [47, 48].

Our study also had some limitations. One is its short duration, which may have caused underestimation of the effectiveness of folate. An additional limitation of the study may be the dosing. The optimal dose of folic acid needed to improve cognitive function is currently unknown. Although the present study provides some insight, larger dosages may produce different effects than those observed in the current investigation. Lastly, because all participants received donepezil, the effects on AD patients who are not prescribed donepezil must be identified in future studies.

## 5. Conclusions

This small pilot study examined the effect of folic acid supplementation on newly diagnosed patients with AD. Folic acid improved cognition and markers of inflammation. The full effects of folate may lie in its ability to work in concert with donepezil and may lead to improved clinical practices in the treatment of AD.

## Figures and Tables

**Table 1:** Primer sequences of the internal control gene and the target genes for RT-PCR.

Genes	Primer sequence
APP	Forward: CCTACAACAGCAGCCAGTACCCCTG
	Reverse: GACATTCTCTCTCGGTGCTTGGCC

PS1	Forward: GGTCGTGGCTACCATTAAGTC
	Reverse: GCCCACAGTCTCGGTATCTT

PS2	Forward: ACCGCTATGTCTGTAGTGGG
	Reverse: CGCTCCGTATTTGAGGGTCAG

IL-6	Forward: TCTCCACAAGCGCCTTCG
	Reverse: CTCAGGGCTGAGATGCCG

TNF*α*	Forward: TCTTCTCGAACCCCGAGTGA
	Reverse: CCTCTGATGGCACCACCAG

GAPDH	Forward: CCACTCCTCCACCTTTGAC
	Reverse: ACCCTGTTGCTGTAGCCA

**Table 2:** Baseline characteristics of the study population.

Profile	Intervention group (*n* = 61)	Control group (*n* = 60)	*P* value^a^
Demography			
Age at diagnosis, years (mean ± SD)	68.10 ± 8.50	67.63 ± 7.92	0.756
Males, number (%)	33 (54.10)	28 (46.67)	0.414
Education, years (mean ± SD)	10.07 ± 4.08	9.27 ± 4.39	0.302
Lifestyle			
Right-handedness, number (%)	57 (93.44)	53 (88.33)	0.363
Marital status: married, number (%)	50 (81.97)	49 (81.67)	0.966
Living with others, number (%)	60 (98.36)	58 (96.67)	0.619
Smoking, number (%)	12 (19.67)	14 (23.33)	0.624
Consuming alcohol, number (%)	10 (16.39)	12 (20.00)	0.607
BMI (mean ± SD), kg/m^2^	23.25 ± 3.06	23.58 ± 4.28	0.623
Profession: intellectual work, number (%)	32 (52.46)	28 (46.67)	0.524
Family history, number (%)	16 (26.23)	15 (25.00)	0.877
Comorbid disease, *n* (%)	25 (40.98)	26 (43.33)	0.794
Biochemical measures			
Folate (nmol/L) [median (*Q*1, *Q*3)]	11.98 (8.04, 17.68)	10.76 (7.53, 16.37)	0.484
Vitamin B_12_ (pmol/L) [median (*Q*1, *Q*3)]	289.96 (233.88, 406.16)	329.06 (231.86, 455.04)	0.446
Medicine on AD			
AChEI, number (%)	61 (100.00)	60 (100.00)	1.000
Memantine, number (%)	10 (16.39)	13 (15.48)	0.460
MMSE (mean ± SD)	18.56 ± 6.23	17.63 ± 7.77	0.471
ADL (mean ± SD)	32.87 ± 10.88	33.97 ± 13.42	0.622
Folate insufficiency^b^	18 (29.51)	24 (40.00)	0.225

AChEI: acetylcholinesterase inhibitor; AD: Alzheimer's disease; ADL: activities of daily living. Domains of comorbidity include diabetes, hypertension, coronary heart disease, cerebral infarction, cerebral hemorrhage, transient cerebral hemorrhage, cerebral trauma, Parkinson's disease, CO poisoning, tumor, exposure to heavy metals, anxiety, and depression. Results are expressed as proportion, mean ± SD, or median (*Q*1, *Q*3). Comparisons between intervention groups and controls were performed by two-tailed Student's *t*-test (for normally distributed data) and Mann-Whitney test (for skewed distribution). Pearson's chi-square and Fisher's Exact Test were performed for frequencies analysis; *n*: number of individuals. ^a^ *P* < 0.05, significant difference between groups; ^b^based on WHO definitions of folate sufficiency [below 9.06 nmol/L (4 ng/mL)].

**Table 3:** The levels of serum biomarkers parameters at baseline and 6-month follow-up in folic acid and control groups.

Item	Groups	Cases (*n*)	Treatment time		Repeated measures		
			Before treatment	At 6 months	Interaction effect, *P* (*ηp* ^2^)	Time effect, *P* (*ηp* ^2^)	Group effect, *P* (*ηp* ^2^)
Folate (nmol/L)	Intervention	61	11.98 (8.04, 17.68)	25.37 (10.87, 34.54)	0.001 (0.937)	0.000 (0.972)	0.001 (0.933)
	Control	60	10.76 (7.53, 16.37)	10.76 (7.99, 16.99)			

Vitamin B_12_ (pmol/L)	Intervention	61	289.96 (233.88, 406.16)	289.22 (240.89, 397.68)	0.627 (0.077)	0.854 (0.054)	0.488 (0.106)
	Control	60	329.06 (231.86, 455.04)	328.33 (225.58, 445.82)			

Hcy (*µ*mol/L)	Intervention	61	17.55 ± 7.25	12.25 ± 6.93	0.096 (0.385)	0.000 (0.994)	0.001 (0.903)
	Control	60	19.16 ± 7.34	16.73 ± 6.13			

SAM (nmol/L)	Intervention	61	78.53 ± 35.02	99.04 ± 33.14	0.012 (0.712)	0.055 (0.485)	0.092 (0.391)
	Control	60	83.81 ± 32.11	81.06 ± 29.55			

SAH (nmol/L)	Intervention	61	127.20 ± 45.48	97.24 ± 52.19	0.051 (0.499)	0.004 (0.826)	0.078 (0.422)
	Control	60	126.19 ± 54.33	120.39 ± 38.40			

SAM/SAH	Intervention	61	0.57 (0.49, 0.75)	1.27 (0.69, 1.85)	0.000 (0.999)	0.000 (0.991)	0.004 (0.882)
	Control	60	0.61 (0.51, 0.80)	0.62 (0.53, 0.78)			

A*β*_40_ (pg/mL)	Intervention	61	30.11 (11.98, 63.39)	22.72 (8.83, 54.48)	0.003 (0.843)	0.827 (0.048)	0.029 (0.592)
	Control	60	32.94 (12.39, 60.23)	46.47 (16.33, 92.43)			

A*β*_42_ (pg/mL)	Intervention	61	53.88 (35.72, 115.56)	45.17 (31.11, 68.22)	0.306 (0.175)	0.009 (0.754)	0.021 (0.642)
	Control	60	42.32 (19.62, 76.66)	35.67 (23.66, 56.01)			

A*β*_42_/A*β*_40_	Intervention	61	2.12 (1.07, 3.40)	1.86 (0.81, 4.98)	0.029 (0.594)	0.136 (0.319)	0.001 (0.940)
	Control	60	1.46 (0.56, 3.75)	0.85 (0.29, 1.80)			

IL-6 (pg/mL)	Intervention	61	8.12 (5.93, 12.61)	9.94 (6.87, 13.30)	0.334 (0.161)	0.398 (0.134)	0.004 (0.834)
	Control	60	10.23 (6.12, 16.98)	10.88 (6.77, 18.71)			

TNF*α* (pg/mL)	Intervention	61	62.88 (26.53, 277.39)	56.08 (21.06, 252.61)	0.021 (0.638)	0.504 (0.102)	0.308 (0.174)
	Control	60	85.89 (18.81, 261.05)	112.42 (26.37, 254.75)			

MMSE	Intervention	61	18.56 ± 6.23	18.72 ± 6.56	0.041 (0.538)	0.167 (0.281)	0.274 (0.193)
	Control	60	17.63 ± 7.77	16.80 ± 8.26			

ADL	Intervention	61	32.87 ± 10.88	32.93 ± 10.93	0.895 (0.052)	0.698 (0.067)	0.615 (0.079)
	Control	60	33.97 ± 13.42	34.10 ± 14.15			

Biochemical variables are presented as mean ± SD or median (quartiles). Results are expressed as proportion, mean ± SD, or median (*Q*1, *Q*3). Hcy, SAM, SAH, MMSE, and ADL, which were normally distributed, and folate, vitamin B_12_, SAM/SAH, A*β*_40_, A*β*_42_, A*β*_42_/A*β*_40_, IL-6, and TNF*α*, which were normally distributed after logarithmic transformation, were analyzed by repeated-measures ANOVA. *ηp* ^2^ describes the percentage of variance explained in the dependent variable by a predictor variable.

**Table 4:** The mRNA levels of genes related to serum biomarker parameters at baseline and 6-month follow-up in folic acid and control groups.

Item	Groups	Cases (*n*)	Treatment time		Repeated measures		
			Before treatment	At 6 months	Interaction effect, *P* (*ηp* ^2^)	Time effect, *P* (*ηp* ^2^)	Group effect, *P* (*ηp* ^2^)
PS1-mRNA	Intervention	24	50.71 (28.66, 128.88)	43.44 (21.81, 85.86)	0.017 (0.676)	0.008 (0.781)	0.000 (0.988)
	Control	24	37.16 (30.98, 66.76)	143.10 (107.14, 241.16)			

PS2-mRNA	Intervention	24	21.34 (2.87, 70.20)	4.68 (1.58, 22.78)	0.055 (0.488)	0.823 (0.056)	0.775 (0.059)
	Control	24	5.86 (2.09, 0.29)	9.50 (5.76, 13.77)			

APP-mRNA	Intervention	24	14.56 (6.99, 30.98)	20.68 (8.79, 24.40)	0.358 (0.149)	0.970 (0.050)	0.064 (0.458)
	Control	24	17.79 (8.12, 73.90)	19.88 (9.82, 86.05)			

IL-6-mRNA	Intervention	24	0.43 (0.21, 0.89)	0.28 (0.17, 0.47)	0.101 (0.373)	0.195 (0.251)	0.080 (0.417)
	Control	24	0.26 (0.12, 0.51)	0.22 (0.11, 0.53)			

TNF*α*-mRNA	Intervention	24	6.20 (3.00, 11.12)	2.86 (1.44, 5.73)	0.044 (0.527)	0.091 (0.393)	0.520 (0.097)
	Control	24	5.43 (2.19, 8.51)	5.50 (3.35, 8.48)			

Variables are presented as median (*Q*1, *Q*3). Serum biomarkers, which were normally distributed after logarithmic transformation, are analyzed by repeated-measures ANOVA. *ηp* ^2^ describes the percentage of variance explained in the dependent variable by a predictor variable.

## Abbreviations

AChEI: Acetylcholinesterase inhibitor
AD: Alzheimer's disease
ADL: Activities of daily living
APP: Amyloid precursor protein
A*β*: Amyloid beta
BMI: Body mass index
CSF: Cerebrospinal fluid
EDTA: Ethylenediaminetetraacetic acid
Hcy: Homocysteine
HPLC: High-performance liquid chromatography
ITT: Intention-to-treat
IL-6: Interleukin-6
LPS: Lipopolysaccharide
MCI: Mild cognitive impairment
MMSE: Mini-Mental State Examination
PS: Presenilin
RCT: Randomized controlled trial
RT-PCR: Real-time polymerase chain reaction
SAM: S-Adenosylmethionine
SAH: S-Adenosylhomocysteine
SD: Standard deviation
TNF*α*: Tumor necrosis factor alpha.

## References

[1] Evans D. A., Funkenstein H. H., Albert M. S. (1989). Prevalence of Alzheimer's disease in a community population of older persons, higher than previously reported. *The Journal of the American Medical Association*.

[2] Chan K. Y., Wu J. J., Liu L. (2013). Epidemiology of Alzheimer's disease and other forms of dementia in China, 1990–2010: a systematic review and analysis. *The Lancet*.

[3] Masters C. L., Cappai R., Barnham K. J., Villemagne V. L. (2006). Molecular mechanisms for Alzheimer's disease: implications for neuroimaging and therapeutics. *Journal of Neurochemistry*.

[4] Chouliaras L., Mastroeni D., Delvaux E. (2013). Consistent decrease in global DNA methylation and hydroxymethylation in the hippocampus of Alzheimer's disease patients. *Neurobiology of Aging*.

[5] Gezen-Ak D., Dursun E., Hanağası H. (2013). BDNF, TNF*α*, HSP90, CFH, and IL-10 serum levels in patients with early or late onset Alzheimer's disease or mild cognitive impairment. *Journal of Alzheimer's Disease*.

[6] Das U. N. (2008). Folic acid and polyunsaturated fatty acids improve cognitive function and prevent depression, dementia, and Alzheimer's disease—but how and why?. *Prostaglandins Leukotrienes and Essential Fatty Acids*.

[7] Chen H., Liu S., Ji L. (2015). Associations between Alzheimer’s disease and blood homocysteine, vitamin B12, and folate: a case-control study. *Current Alzheimer Research*.

[8] Clarke R., Smith A. D., Jobst K. A., Refsum H., Sutton L., Ueland P. M. (1998). Folate, vitamin B12, and serum total homocysteine levels in confirmed Alzheimer disease. *Archives of Neurology*.

[9] Postiglione A., Milan G., Ruocco A., Gallotta G., Guiotto G., Di Minno G. (2001). Plasma folate, vitamin B_12_, and total homocysteine and homozygosity for the C677T mutation of the 5,10-methylene tetrahydrofolate reductase gene in patients with Alzheimer's dementia. A case-control study. *Gerontology*.

[10] McMahon J. A., Green T. J., Skeaff C. M., Knight R. G., Mann J. I., Williams S. M. (2006). A controlled trial of homocysteine lowering and cognitive performance. *The New England Journal of Medicine*.

[11] Durga J., van Boxtel M. P., Schouten E. G. (2007). Effect of 3-year folic acid supplementation on cognitive function in older adults in the FACIT trial: a randomised, double blind, controlled trial. *The Lancet*.

[12] Zheng M., Zhang M., Yang J. (2014). Relationship between blood levels of methyl donor and folate and mild cognitive impairment in Chinese patients with type 2 diabetes: a case-control study. *Journal of Clinical Biochemistry and Nutrition*.

[13] Jack C. R., Albert M. S., Knopman D. S. (2011). Introduction to the recommendations from the National Institute on Aging-Alzheimer's Association workgroups on diagnostic guidelines for Alzheimer's disease. *Alzheimer's and Dementia*.

[14] Feng F., Han X. Q., Chen J., Shang L., Li J. (2004). Application of the activities of daily living rating scale in screening dementia. *J Clin Psychol Med*.

[15] Papandreou D., Mavromichalis I., Makedou A., Rousso I., Arvanitidou M. (2006). Total serum homocysteine, folate and vitamin B12 in a Greek school age population. *Clinical Nutrition*.

[16] Quinlivan E. P. (2008). In vitamin B12 deficiency, higher serum folate is associated with increased homocysteine and methylmalonic acid concentrations. *Proceedings of the National Academy of Sciences of the United States of America*.

[17] Poirier L. A., Wise C. K., Delongchamp R. R., Sinha R. (2001). Blood determinations of S-adenosylmethionine, S-adenosylhomocysteine, and homocysteine: correlations with diet. *Cancer Epidemiology Biomarkers and Prevention*.

[18] Sun Z. Q. (2013). *Medical Statistics*.

[19] Ballard C., Margallo-Lana M., Juszczak E. (2005). Quetiapine and rivastigmine and cognitive decline in Alzheimer's disease: randomised double blind placebo controlled trial. *The British Medical Journal*.

[20] Berry R. J., Li Z., Erickson J. D. (1999). Prevention of neural-tube defects with folic acid in China. *The New England Journal of Medicine*.

[21] Schmidt R., Schmidt H., Curb J. D., Masaki K., White L. R., Launer L. J. (2002). Early inflammation and dementia: a 25-year follow-up of the Honolulu-Asia Aging Study. *Annals of Neurology*.

[22] Engelhart M. J., Geerlings M. I., Meijer J. (2004). Inflammatory proteins in plasma and the risk of dementia: the rotterdam study. *Archives of Neurology*.

[23] Das S., Basu A. (2008). Inflammation: a new candidate in modulating adult neurogenesis. *Journal of Neuroscience Research*.

[24] Marsland A. L., Gianaros P. J., Abramowitch S. M., Manuck S. B., Hariri A. R. (2008). Interleukin-6 covaries inversely with hippocampal grey matter volume in middle-aged adults. *Biological Psychiatry*.

[25] McAfoose J., Baune B. T. (2009). Evidence for a cytokine model of cognitive function. *Neuroscience and Biobehavioral Reviews*.

[26] Chaney M. O., Baudry J., Esh C. (2003). A*β*, aging, and Alzheimer's disease: a tale, models, and hypotheses. *Neurological Research*.

[27] De Strooper B., König G. (1999). Alzheimer's disease: a firm base for drug development. *Nature*.

[28] Selkoe D. J. (2000). Toward a comprehensive theory for Alzheimer's disease. Hypothesis: Alzheimer's disease is caused by the cerebral accumulation and cytotoxicity of amyloid *β*-protein. *Annals of the New York Academy of Sciences*.

[29] Findeis M. A. (2007). The role of amyloid *β* peptide 42 in Alzheimer's disease. *Pharmacology and Therapeutics*.

[30] Bentahir M., Nyabi O., Verhamme J. (2006). Presenilin clinical mutations can affect *γ*-secretase activity by different mechanisms. *Journal of Neurochemistry*.

[31] Fukuyama R., Mizuno T., Mizuno T. (2000). Age-dependent change in the levels of A*β*40 and A*β*42 in cerebrospinal fluid from control subjects, and a decrease in the ratio of A*β*42 to A*β*40 level in cerebrospinal fluid from Alzheimer's disease patients. *European Neurology*.

[32] Hansson O., Zetterberg H., Buchhave P. (2007). Prediction of Alzheimer's disease using the CSF A*β*42/A*β*40 ratio in patients with mild cognitive impairment. *Dementia and Geriatric Cognitive Disorders*.

[33] Yaffe K., Weston A., Graff-Radford N. R. (2011). Association of plasma *β*-amyloid level and cognitive reserve with subsequent cognitive decline. *The Journal of the American Medical Association*.

[34] Bottiglieri T., Hyland K. (1994). S-adenosylmethionine levels in psychiatric and neurological disorders: a review. *Acta Neurologica Scandinavica*.

[35] Morrison L. D., Smith D. D., Kish S. J. (1996). Brain S-adenosylmethionine levels are severely decreased in Alzheimer's disease. *Journal of Neurochemistry*.

[36] Tchantchou F., Graves M., Ortiz D., Chan A., Rogers E., Shea T. B. (2006). S-adenosyl methionine: a connection between nutritional and genetic risk factors for neurodegeneration in Alzheimer's disease. *Journal of Nutrition, Health and Aging*.

[37] Trolin C. G., Regland B., Oreland L. (1995). Decreased methionine adenosyltransferase activity in erythrocytes of patients with dementia disorders. *European Neuropsychopharmacology*.

[38] Bottiglieri T., Godfrey P., Flynn T., Carney M. W. P., Toone B. K., Reynolds E. H. (1990). Cerebrospinal fluid S-adenosylmethionine-in depression and dementia: effects of treatment with parenteral and oral S-adenosylmethionine. *Journal of Neurology, Neurosurgery and Psychiatry*.

[39] Pfalzer A. C., Choi S.-W., Tammen S. A. (2014). S-adenosylmethionine mediates inhibition of inflammatory response and changes in DNA methylation in human macrophages. *Physiological Genomics*.

[40] Gobejishvili L., Avila D. V., Barker D. F. (2011). *S*-adenosylmethionine decreases lipopolysaccharide-induced phosphodiesterase 4B2 and attenuates tumor necrosis factor expression via cAMP/protein kinase A pathway. *Journal of Pharmacology and Experimental Therapeutics*.

[41] Purohit V., Abdelmalek M. F., Barve S. (2007). Role of S-adenosylmethionine, folate, and betaine in the treatment of alcoholic liver disease: summary of a symposium. *American Journal of Clinical Nutrition*.

[42] Sharma B., Singh N. (2012). Salutary effect of NF*κ*B inhibitor and folacin in hyperhomocysteinemia-hyperlipidemia induced vascular dementia. *Progress in Neuro-Psychopharmacology and Biological Psychiatry*.

[43] Connelly P. J., Prentice N. P., Cousland G., Bonham J. (2008). A randomised double-blind placebo-controlled trial of folic acid supplementation of cholinesterase inhibitors in Alzheimer's disease. *International Journal of Geriatric Psychiatry*.

[44] Chen T.-F., Huang R.-F. S., Lin S.-E., Lu J.-F., Tang M.-C., Chiu M.-J. (2010). Folic acid potentiates the effect of memantine on spatial learning and neuronal protection in an Alzheimer's disease transgenic model. *Journal of Alzheimer's Disease*.

[45] Shea T. B., Lyons-Weiler J., Rogers E. (2002). Homocysteine, folate deprivation and Alzheimer neuropathology. *Journal of Alzheimer's Disease*.

[46] Creeley C. E., Wozniak D. F., Nardi A., Farber N. B., Olney J. W. (2008). Donepezil markedly potentiates memantine neurotoxicity in the adult rat brain. *Neurobiology of Aging*.

[47] Atri A., Shaughnessy L. W., Locascio J. J., Growdon J. H. (2008). Long-term course and effectiveness of combination therapy in Alzheimer disease. *Alzheimer Disease and Associated Disorders*.

[48] Lopez O. L., Becker J. T., Wahed A. S. (2009). Long-term effects of the concomitant use of memantine with cholinesterase inhibition in Alzheimer disease. *Journal of Neurology, Neurosurgery and Psychiatry*.

